# Supplementary material for: Choriocapillaris flow deficit in a pachychoroid spectrum disease using en face optical coherence tomography angiography averaging
Source: PLoS One. 2022 Sep 12;17(9):e0271747. doi: 10.1371/journal.pone.0271747 (PMC9467345; doi:10.1371/journal.pone.0271747)
Supplement: Attachment — Submitted filename: Point by point response.docx [file pone.0271747.s002.docx]

Reviewer #1: Major comments
Authors investigated the choriocapillaris changes associated with pachychoroid pigment epitheliopathy (PPE) in comparison with healthy eyes. Authors found that the blood flow area of the choriocapillaris decreased diffusely within the macular area in PPE eyes compared to control eyes, and the choriocapillaris flow deficit was not necessarily related to pachyvessel location.

This study shows that choriocapillaris changes in PPE eyes were not directly caused by the pachyvessels. This is an interesting finding. The definition of pachyvessel is not clear. Authors stated that “Pachyvessels were defined as dilated choroidal venous veins that continuously extended from the dilated vortex vein, could be distinguished by their relatively larger caliber in comparison with other choroidal veins in other quadrants, and showed asymmetry of upper and lower vortex veins.” This is a subjective and vague definition, and it is difficult for other researchers to follow this study. This is part should be stated in the limitation section.

A. The presence of pachyvessels was evaluated by using choroidal wide-angle en face OCT images (12 × 12 mm) and wide-angle IA. The presence of pachyvessels in each case was determined in agreement by two retinal specialists. (line221)

As suggested by the reviewer, we added the followings in the limitation section.

“Fourth, the definition of pachyvessel is subjective and vague. No objective or quantitative definition has been established so far. In the current study, the pachyvesssel was diagnosed by two retinal specialists with multimodal images.” (line 443)

In addition, this study does not pay attention to the distance between pachyvessel and PRE. Because PRE abnormality, PRE detachment, or choroidal neovascularization tend to occur on or near the site where this distance is very small, this distance is an important factor when evaluating PPE. This may be why there was no significant difference in Table 3. If authors ignored this parameter, this part also should be stated in the limitation section.

A. As the reviewer suggested, we mentioned the parameter and added or revised the following in the methods, Table3, Results, and limitation section, respectively.

“**Measurement of the distance between RPE and choroidal venous vein**

We evaluated the distance between RPE and choroidal venous vein. We manually measured the distance at the center of the fovea, 1mm superior side from the fovea, 1mm inferior side from the fovea, 1mm nasal side from the fovea, and 1mm temporal side from the fovea by using a built-in caliber tool. We used vertical and horizontal B-scans through the fovea, and averaged each distance.” (line 240)

“The two groups showed no significant differences in number of flow voids, total flow void area, average size of the flow voids, and SFCT after adjusting for age and sex. The distance between RPE and choroidal venous vein in pachyessel group was significantly shorter than that in non-pachyvessel group.” (line 318)

Minor comments
1. Eye of PPE were fellow eyes of CSC or normal eyes? Please add information of the patients of PPE.

A. As suggested by the reviewer, we added the following in methods section.

“Eyes of PPE were fellow eyes of PNV, CSC or PPE. If both eyes were eligible, the right eye was included.” (line 153)

2. Table 2 and Table 3: “p values adjusted for age and sex.”
Was this comparison possible using un-paired t-test, as stated in the Method.

A. Thank you for helpful advice. We used partial correlation for adjusting for age and sex. The followings were added in statistical analysis in methods section.

“Partial correlation was used for adjusting for age and sex.” (line 263)

3. Table 3: Please add the information of choroidal thickness.

A. We added the information of choroidal thickness in Table 3.

Reviewer #2: Thank you for inviting me to review the manuscript entitled, "Choriocapillaris flow deficit in a pachychoroid spectrum disease using en face optical coherence tomography angiography averaging."

Comments:
1. This study included the eyes with PPE and normal control eyes. However, the detailed information about the contralateral eyes were not provided. In terms of PPE, the contralateral eyes had no abnormalities or only PPE? If so, performing dye angiography sounds ethically problematic. Were there any exudative abnormalities in the contralateral eyes, such as CSC, PNV, pr PCV? In terms of normal control eyes, were there any ocular diseases in the contralateral eyes? Furthermore, the status of contralateral eyes affected the results?

A. As the reviewer suggested, we provided the detailed information about the contralateral eyes of PPE eyes. The following were added in participants in methods section.

“Eyes of PPE were fellow eyes of PNV, CSC or PPE. If both eyes were eligible, the right eye was included.” (line 153)

We mentioned about the information of normal control eyes in participants in methods section.

“We recruited healthy eyes of healthy volunteers and unaffected fellow eyes of patients with unilateral retinal diseases (e.g., epiretinal membrane, vitreomacular traction syndrome) [16]. In the current study, subjects with the systemic conditions like diabetes mellitus or those with poorly controlled hypertension were excluded.” (line 154)

We analyzed whether the status of contralateral eyes affected the results.

“Fellow eyes of eyes with PPE were PNV: 17 eyes, CSC: 11 eyes, PPE: 4 eyes.

In PNV eyes, the mean number of flow voids were significantly smaller than those in CSC eyes (1432 ± 147 vs 1592 ± 111; p = 0.018). In PNV eyes, the mean average size of the flow voids was significantly larger than those in CSC eyes (842 ± 144 vs 704 ± 76; p = 0.041). However, there were no significant differences in the total flow void area among 3 groups.” (line308)

|  | PNV (n=17) | CSC (n=11) | PPE (n=4) | Pa | Pb | Pc |
| --- | --- | --- | --- | --- | --- | --- |
| Number of flow voids | 1432 ± 147 | 1592 ± 111 | 1397 ± 116 | 0.018 | 1.0 | 0.068 |
| Total flow void area | 1.21 ± 0.18 | 1.10 ± 0.13 | 1.08 ± 0.20 | 0.294 | 0.498 | 0.997 |
| Average size of the flow voids | 842 ± 144 | 704 ± 76 | 802 ± 171 | 0.041 | 1.0 | 0.684 |

Pa: PNV vs CSC　　Pb: PNV vs PPE　　Pc: CSC vs PPE (Bonferroni correction)

2. Please demonstrate more information about the systemic conditions that might influence the results (i.e, hypertension and diabetes) should be mentioned. Otherwise, some comments should be included as the limitation of the study.

A. As the reviewer suggested, we added the followings in methods section.

“In the current study, subjects with the systemic conditions like diabetes mellitus or those with poorly controlled hypertension were excluded.” (line 156)
